# Diabetes Device Use and Glycemic Control among Youth with Type 1 Diabetes: A Single-Center, Cross-Sectional Study

**DOI:** 10.1155/2018/5162162

**Published:** 2018-07-29

**Authors:** Khalid Sheikh, Sara K. Bartz, Sarah K. Lyons, Daniel J. DeSalvo

**Affiliations:** ^1^University of Houston, Houston, TX, USA; ^2^Baylor College of Medicine/Texas Children's Hospital, Houston, TX, USA

## Abstract

**Aim:**

The purpose of this cross-sectional study was to determine the rates of diabetes device use (insulin pump and continuous glucose monitor (CGM)) and association with glycemic control in youth with type 1 diabetes in a large, diverse pediatric center.

**Methods:**

Demographic and clinical data were obtained from 1992 patients who met the eligibility criteria (age < 26 years, diabetes duration ≥ 1 year, and ≥1 clinic visit in the preceding 12 months). Statistical analyses assessed the likelihood of device use based on demographic characteristics and the association between device use and glycemic control based on most recent hemoglobin A1c (HbA1c).

**Results:**

Mean age was 13.8 ± 4.2 years, 50.7% were female, diabetes duration was 6.2 ± 4 years, and mean HbA1c was 8.7 ± 1.8%. Overall, 38.2% of patients were on pump therapy and 18.5% were on CGM. Patients who were non-Hispanic (NH) white, privately insured, and with primary English-speaking parent(s) had higher rates of insulin pump use, as well as CGM use (*P* < 0.001 for both). Female patients had higher rates of pump use only (*P* < 0.01). Private health insurance, NH white race/ethnicity, and CGM use were each associated with lower HbA1c (*P* = 0.03, <0.001, and <0.008, resp.).

**Conclusion:**

At a large, diverse, pediatric diabetes center, disparities in diabetes device use were present across sex, race/ethnicity, health insurance coverage, and primary language of parent(s). CGM use was associated with lower HbA1c. Quality improvement efforts are underway to ensure improved access, education, and clinical programs for advanced diabetes devices for T1D patients.

## 1. Introduction

Intensive treatment of type 1 diabetes (T1D) leads to improved glycemic control resulting in reduced risk of microvascular complications [1–3]. Current American Diabetes Association (ADA) recommendations for pediatric patients with T1D are aimed at achieving near normal glycemia (HbA1c < 7.5% for children and <7.0% for young adults) while avoiding severe hypoglycemic events [4]; however, the vast majority of youth and young adults with T1D are not meeting glycemic targets. Fewer than 25% of pediatric patients in the T1D Exchange Registry, a US-based registry comprised of over 26,000 individuals with T1D, achieve the ADA-recommended HbA1c target [5].

Insulin pumps and continuous glucose monitors (CGM) are advanced diabetes management devices that may lead to improved glycemic control compared to traditional insulin injections with self-monitoring of blood glucose (SMBG) [6–11]. Compared to injections, insulin pump therapy offers a more physiologic method of insulin delivery by simulating the normal diurnal pattern of basal insulin secretion in conjunction with prandial or correction boluses [12]. CGM is an emerging technology that provides a continuous measure of interstitial fluid glucose levels to provide real-time trends and alerts to glucose excursions [13]. Despite their potential benefit for improving glycemic control, uptake of these technologies has been limited with 60% of T1D Exchange Registry participants using an insulin pump and a mere 11% using CGM [5].

In this study, we evaluated the rates of advanced diabetes device use and association with glycemic control among pediatric and young adult patients with T1D at Texas Children's Hospital (TCH)—a large, academic, tertiary urban hospital with a diverse patient population.

## 2. Methods

The study was approved by the Baylor College of Medicine Institutional Review Board. We deployed the EPIC® electronic medical record population health management system for the Texas Children's Hospital diabetes clinic patient registry to generate a comprehensive data report to perform a cross-sectional analysis. Recorded variables included age, sex, race/ethnicity, diabetes type, date of diagnosis, insurance coverage, primary spoken language of parent(s), insulin management category, use of CGM, and most recent HbA1c measurement. Medical insurance was classified as public for patients with coverage through Medicaid or Children's Health Insurance Program (CHIP). Eligibility criteria included patients < 26 years of age with a clinical diagnosis of T1D of at least 1-year duration with at least one diabetes clinic visit between July 1, 2015, and June 30, 2016.

### 2.1. Statistical Analysis

Fisher's exact test was used to compare the distribution of patient characteristics between those who use technology (CGM and/or insulin pump) and those who do not. The multiple logistic regression model including sex, primary language of parent(s), insurance type (public or private), and race/ethnicity was used to estimate the odds of not using pump therapy and not using CGM. A general linear model (least square means) was used to analyze significant associations between the predictor variables and HbA1c. Unadjusted HbA1c data was analyzed by *t*-test for 2-group comparisons and analysis of variance (ANOVA) for multiple-group comparisons. Data analyses were performed using SAS version 9.4 (2011 SAS Institute Inc., Cary, NC). Two-sided *P* values < 0.05 were considered statistically significant. Data are presented as mean ± standard deviation unless otherwise specified.

## 3. Results

A total of 1992 T1D patients met the criteria, with 50.7% female, mean age of 13.8 ± 4.2 years, duration of diabetes of 6.2 ± 4.0 years, and HbA1c of 8.7 ± 1.8% (Table 1).

Overall, 761 (38.2%) patients were using an insulin pump and 369 (18.5%) were using CGM for diabetes management. Females were more likely to use insulin pumps than males (*P* < 0.01), while rates of CGM use were similar between males and females (*P* = 0.06). Non-Hispanic (NH) whites were more likely to use insulin pumps (*P* < 0.001) and CGM (*P* < 0.001) than minority patients. Patients with private health insurance were more likely to use insulin pumps (*P* < 0.001) and CGM (*P* < 0.001) than those with public health insurance or no insurance. Patients with English-speaking parent(s) were more likely to use insulin pumps (*P* < 0.001) and CGM (*P* < 0.001) than those with Spanish-speaking parent(s) (Table 2).

**Figure 1:**
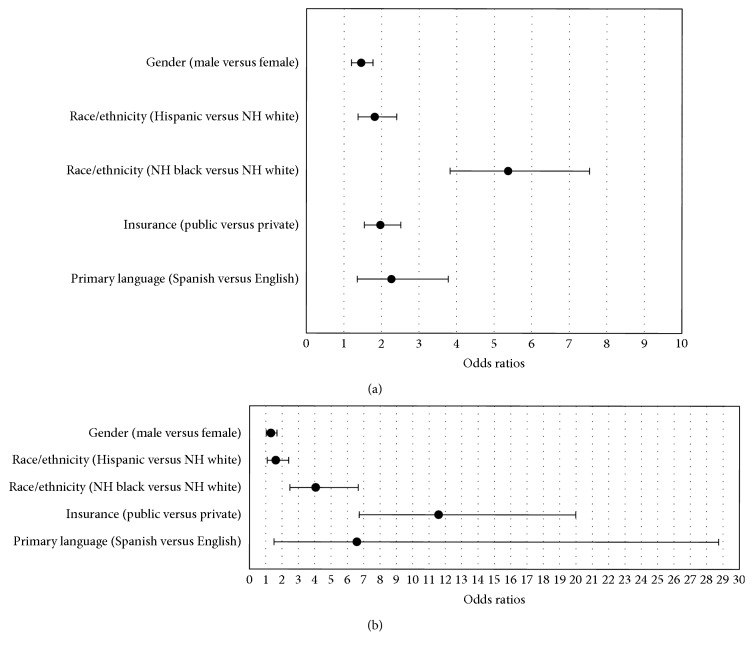
(a) Odds ratios of not using an insulin pump (95% confidence intervals). (b) Odds ratios of not using CGM (95% confidence intervals).

In multiple logistic regression modeling, all variables maintained significant associations with pump use after simultaneously adjusting for the other variables in the model. The odds of not using a pump were greater among males (OR: 1.46; 95% CI: 1.20, 1.77), patients with Spanish-speaking parent(s) (OR: 2.27; 95% CI: 1.36, 3.78), and those with public health insurance (OR: 1.97; 95% CI: 1.54, 2.54). The odds of not using a pump were also greater among Hispanics (OR: 1.82; 95% CI: 1.37, 2.40) and NH blacks (OR: 5.37; 95% CI: 3.83, 7.54) than among NH whites (Figure 1(a)).

Similarly, all variables maintained statistically significant associations with CGM use after simultaneously adjusting for the other variables in the model. The odds of not using CGM were greater among males (OR: 1.32; 95% CI: 1.03, 1.69), patients with Spanish-speaking parent(s) (OR: 6.59; 95% CI: 1.51, 28.73), and those with public insurance (OR: 11.59; 95% CI: 6.72, 19.99). The odds of not using CGM were also greater among Hispanics (OR: 1.62; 95% CI: 1.09, 2.40) and NH blacks (OR: 4.06; 95% CI: 2.48, 6.66) than among NH whites (Figure 1(b)).

In the general linear model (least square means) analyzing the association between predictor variables and HbA1c, private health insurance (*P* = 0.003) and NH whites (*P* < 0.001) were associated with lower HbA1c estimates after simultaneously adjusting for all variables in the model. Neither sex nor primary language of parents was found to have a difference in HbA1c estimate. CGM use was associated with lower HbA1c estimate (*P* < 0.008), but pump use was not (*P* = 0.295) (Table 3).

**Figure 2:**
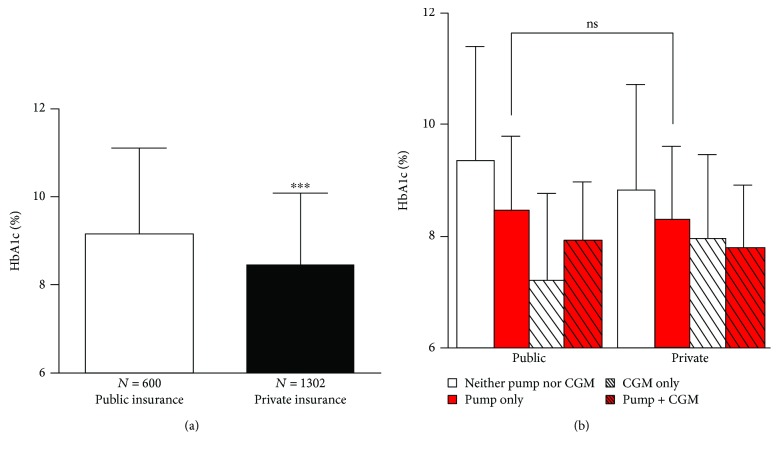
(a) Unadjusted HbA1c data by insurance type. (b) Unadjusted HbA1c data for insurance type analyzed by diabetes treatment regimen stratified by pump and/or CGM use. ∗∗∗ indicates *P* < 0.001; ns indicates not statistically significant.

In the analysis of unadjusted HbA1c data, publicly insured patients had higher mean HbA1c than privately insured patients (9.1 ± 2.0% versus 8.4 ± 1.6%, *P* ≤ 0.001); however, this difference was not seen between publicly insured pump users and privately insured pump users (8.5 ± 1.5% versus 8.3 ± 1.4%, *P* = 0.279) (Figure 2).

## 4. Discussion

This single-center, cross-sectional study analyzed the associations between diabetes device use and demographic factors in pediatric and young adult patients with T1D and between device use and glycemic control. In our large, diverse, pediatric diabetes center, differences in diabetes device use were present for sex, race/ethnicity, health insurance coverage, and primary language of parent(s).

Disparities in device use and glycemic control were found between Hispanic and NH black patients compared to NH white patients. These racial/ethnic differences are in line with studies reporting lower rates of device use for Hispanic and NH black patients compared to NH white patients [14–16]. The previous study suggested that racial/ethnic disparities in diabetes device use may be perpetuated by subconscious racial stereotyping by providers assessing minority patients' preparedness for diabetes devices [16].

Disparities in rates of device use and glycemic control were also present between patients with Spanish-speaking parent(s) compared to those with English-speaking parent(s). The previous study reported language barrier as a contributing factor to low diabetes device use, possibly due to inadequate education regarding the benefits of these technologies [17]. Notably, patients who are Hispanic and have Spanish-speaking parents face two potential barriers (minority status and language) to diabetes device uptake. In effort to improve rates of diabetes device use, our diabetes center has recently developed comprehensive education materials, available in both Spanish and English. The clinic also employs bilingual certified diabetes educators proficient in Spanish to deliver the education to Spanish-speaking families.

Previous studies have reported that socioeconomic status impacts diabetes device use due to financial concerns of families [15, 18]. The difference in rates of diabetes device use between patients with private and public health insurance at our institution was striking. In Texas, public insurance programs including Medicaid and CHIP provide comprehensive coverage for insulin pump therapy but not currently for CGM devices. Therefore, the large disparity in pump use between publicly and privately insured patients was unexpected and suggests that affordability may not be the primary barrier to diabetes device use. The disparities in device use may be due to unmeasured variables such as patient preference or provider biases in interpreting patients' device preparedness. The recent study reported that patients with private insurance have better glycemic control than patients with public insurance [19]. Results from the analysis of our unadjusted data suggest that insulin pump use may mitigate the difference in HbA1c found between patients on public and private insurance; however, the cross-sectional study design does not determine the causality of pump use in lowering HbA1c.

Our finding that CGM use was associated with improved glycemic control (lower mean HbA1c) is consistent with numerous studies [20–24]. CGM provides patients with a real-time view of the glucose level and trends to augment diabetes treatment decisions on a frequent basis to optimize glycemic control. Additionally, downloading CGM data provides a comprehensive pattern of glycemic trends, allowing providers to make more informed adjustments in insulin regimens [13]. Our findings suggest that CGM use may be more effective in improving glycemic control (lower HbA1c) than insulin pump therapy alone. This suggests that if choosing between one device and the other, CGM may be preferred initially. The introduction of two devices at once may add to the overwhelming feeling in diabetes management, especially in newly diagnosed patients [24, 25], so introducing patients to CGM first may lead to improvements in glycemic control while limiting burden of disease on the patient and family. Eventually transitioning patients to sensor-augmented pump therapy may allow patients to benefit from both devices.

Our study has multiple limitations. A major limitation is its cross-sectional design, which does not allow determination of causality of device use with glycemic control. Although we found a correlation between CGM use and lower HbA1c, we were unable to measure the effect of wear time on CGM efficacy. This is a notable limitation since previous studies report that duration of CGM is positively related to reduction in HbA1c [9, 22, 26, 27]. An additional limitation is that our analyses did not include parental income or the level of education, which are important factors in diabetes device use [14–16].

Our findings reveal a myriad of potential barriers in utilizing diabetes devices that may assist patients in achieving optimal glycemic control. Many patients face multiple barriers in device uptake (i.e., minority status, insurance coverage, and language), placing them at further disadvantage in initiating pump therapy and/or CGM. Innovative quality improvement efforts, clinical programs, and research interventions must be implemented to overcome potential barriers to diabetes device use and thereby allow more patients to achieve improved glycemic control.

## 5. Conclusion

Differences in rates of diabetes device use were present across sex, race/ethnicity, health insurance coverage, and primary spoken language of parents. The use of CGM was associated with improved glycemic control (lower HbA1c), more so than insulin pump therapy. Strategies to effectively increase and utilize advanced diabetes devices among T1D patients of all race/ethnicities, insurance types, and languages could substantially improve clinical outcomes. To this end, quality improvement efforts are underway at our center to ensure improved access, education, and clinical programs for advanced diabetes devices for T1D patients.

## Figures and Tables

**Table 1:** Demographic characteristics of the 1992 T1D patients.

	*N* (%)
*Sex*	
Male	982 (49.3%)
Female	1010 (50.7%)
*Age*	
2–6 years	70 (3.5%)
6–<13 years	629 (31.6%)
13–<18 years	905 (45.4%)
18–<26 years	388 (19.5%)
*Race/ethnicity*	
Non-Hispanic white	1043 (52.4%)
Non-Hispanic black	334 (16.8%)
Hispanic	484 (24.3%)
Other	131 (6.6%)
*Insurance type*	
Private	1325 (66.5%)
Public	639 (32.1%)
None	28 (1.4%)
*Primary language of parent(s)*	
English	1836 (92.2%)
Spanish	156 (7.8%)

**Table 2:** Comparison of patient characteristics between those who use diabetes device (pump/CGM) and those who do not.

		Pump			CGM		
		No (%)	Yes (%)	*P* value	No (%)	Yes (%)	*P* value
Sex	Female	585 (47.5)	425 (55.8)	<0.001	806 (49.7%)	204 (55.3%)	0.057
	Male	646 (52.5)	336 (44.2)		817 (50.3%)	165 (44.7%)	

Race/ethnicity	NH white	503 (40.9)	540 (71.0)	<0.001	761 (46.9)	282 (76.4)	<0.001
	NH black	286 (23.2)	48 (6.3)		314 (19.3)	20 (5.4)	
	Hispanic	358 (29.1)	126 (16.6)		444 (27.4)	40 (10.8)	
	Other	84 (6.8)	47 (6.2)		104 (6.4)	27 (7.3)	

Insurance type	None	23 (1.9%)	5 (0.7%)	<0.001	26 (1.6%)	2 (0.5%)	<0.001
	Private	712 (57.8%)	613 (80.6%)		973 (60.0%)	352 (95.4%)	
	Public	496 (40.3%)	143 (18.8%)		624 (38.4%)	15 (4.1%)	

Primary language of parent(s)	English	1098 (89.2%)	738 (97.0%)	<0.001	1469 (90.5%)	367 (99.5%)	<0.001
	Spanish	133 (10.8%)	23 (3.0%)		154 (9.5%)	2 (0.5%)	

**Table 3:** Association between patient characteristics and HbA1C.

		HbA1c estimate	Standard error	Lower	Upper	*P* value
Sex	Female	8.9	0.18	8.6	9.3	*P* = 0.525
	Male	8.9	0.19	8.5	9.2	

Race/ethnicity	NH white	8.5	0.19	8.2	8.9	*P* < 0.001
	NH black	9.6	0.20	9.3	10.0	
	Hispanic	8.9	0.18	8.5	9.2	
	Other	8.6	0.23	8.1	9.0	

Insurance type	None	8.6	0.35	7.9	9.3	*P* = 0.003
	Private	8.9	0.15	8.6	9.2	
	Public	9.2	0.16	8.9	9.5	

Primary language	English	9.0	0.17	8.7	9.3	*P* = 0.234
	Spanish	8.8	0.23	8.4	9.2	

Pump use	No	9.0	0.19	8.6	9.4	*P* = 0.295
	Yes	8.8	0.22	8.4	9.2	

CGM use	No	9.1	0.18	8.7	9.4	*P* = 0.008
	Yes	8.7	0.21	8.3	9.1	

## Data Availability

The authors agree to make our deidentified dataset available by request, immediately upon publication.
